# Determination of Vitamin D Status in a Population of Ecuadorian Subjects

**DOI:** 10.1155/2017/3831275

**Published:** 2017-08-16

**Authors:** G. Maldonado, C. Paredes, R. Guerrero, C. Ríos

**Affiliations:** ^1^Universidad de Especialidades Espíritu Santo, Km. 2.5 Vía la Puntilla, Samborondón, Ecuador; ^2^Centro de Reumatología y Rehabilitación, El Oro y Ambato 1004, Guayaquil, Ecuador

## Abstract

**Introduction:**

Vitamin D is a preprohormone known to play a key role in phosphocalcic metabolism; its main source comes from the synthesis at the skin level by ultraviolet (UV) radiation.

**Objective:**

The purpose of this study was to determine the levels of vitamin D in an Ecuadorian population.

**Materials and Methods:**

Retrospective study of Ecuadorian subjects from the city of Guayaquil, who had an initial study of 25 (OH)-D serum, as the indicator of Vitamin D status, in the period of 2015-2016.

**Results:**

A total of 269 Ecuadorian subjects were analyzed, with a mean age of 54.73 ± 16.58; 85% (229) were females and 15% (41) males; mean vitamin D was 27.29 ± 10.12 ng/dl [6.41–88.74]; 70% of the population showed levels below 30 ng/dL of vitamin D, whereas only 30% (81) had normal values. 69% (185) had levels between 29 and 10 ng/dl and 1% (3) levels below 10 ng/dl. High levels of vitamin D were evidenced in the summer months in relation to the winter months.

**Conclusion:**

It is evident that, despite the location of Ecuador and the intensity of UV rays it receives throughout the year, Ecuadorian subjects have insufficient levels of vitamin D.

## 1. Introduction

Vitamin D is a preprohormone known to play a key role in the metabolism of phosphates and calcium. Its main source is the synthesis by ultraviolet (UV) radiation in the skin [1, 2]. The intensity of UV rays will depend on the height of the sun, latitude, cloudiness, altitude, ozone layer, and soil reflection [3].

Ecuador is located on the equator at a latitude of −0.95, with two seasons: winter (January–April) and summer (May–December). The solar radiation index is 10-11 UV, which is considered as very high radiation levels; however, the general population takes measures to avoid sun exposure which results in insufficient levels of vitamin D. The objective of this study was to determine vitamin D status by measuring serum calcidiol (25 (OH)-D) in an Ecuadorian population that had attended a first consultation at a Rheumatology Center.

## 2. Materials and Methods

Retrospective study of Ecuadorian subjects from the city of Guayaquil, Ecuador, who had an initial study of serum 25 (OH)-D, as the indicator of vitamin D status, from 2015 to 2016.

## 3. Determination of Vitamin D

The 25 (OH)-D serum levels of the patients had been measured in the same laboratory. Samples were analyzed by the chemiluminescence method with an Advia Centaur® assay system. Serum 25 (OH)-D levels are not standardized for each population; however, the following (1.4–6) are considered:

- Ideal: 30–40 ng/dL
- Deficiency: 30–20 ng/dL
- Insufficiency: 20–10 ng/dL
- Severe insufficiency: <10 ng/dL.

The patients were from the city of Guayaquil located in Guayas province, at six meters above sea level and at a latitude of −0.95, with a solar exposure index of 10-11 UV.

### 3.1. Statistical Analysis

The data was analyzed using the statistical program SPSS v 22, with *T*-test of two tails for the quantitative variables and chi-square and logistic regression for the qualitative variables. *P* values below 0.01 were considered statistically significant.

## 4. Results

**Figure 1:**
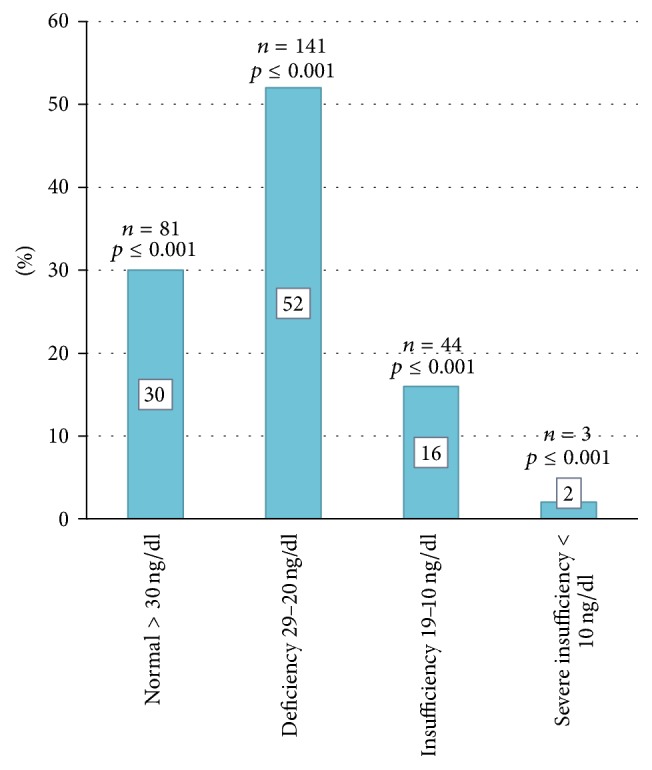
25 (OH)-D levels, as indicator of vitamin D status, in Ecuadorian subjects.

269 Ecuadorian subjects were analyzed, with a mean age of 54.73 ± 16.58; 85%  [229] were female and 15%  [41] male; mean level of 25 (OH)-D was 27.29 ± 10.12 ng/dl [6.41–88.74] (Table 1); 70% of the population showed levels below 30 ng/dL of 25 (OH)-D, whereas only 30% (81) had normal values. 69% (185) had levels between 29 and 10 ng/dl and 1% (3) had levels below 10 ng/dl (Figure 1).

Men had higher levels of 25 (OH)-D than women, but levels in both sexes were deficient with a mean of 27.01 ± 9.72 and 28.89 ± 12.18, respectively.

**Figure 2:**
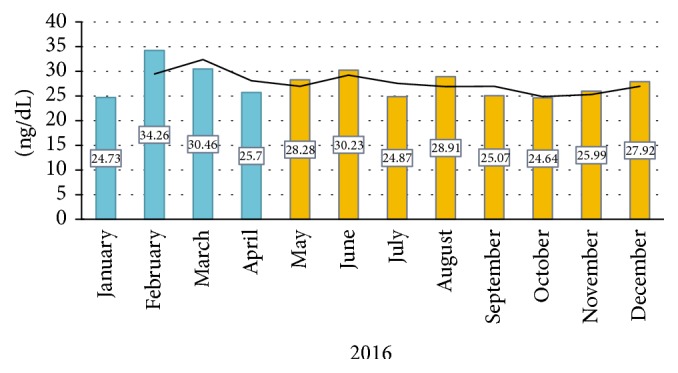
25 (OH)-D levels during the 12 months of the year. Months with the lowest sun exposure: January–April (winter); months with the highest sun exposure: May–December (summer).

Higher levels of 25 (OH)-D were observed in the summer months in relation to the winter months, with February being the month with the highest 25 (OH)-D level 34.26 ± 12.7 (Figure 2).

**Figure 3:**
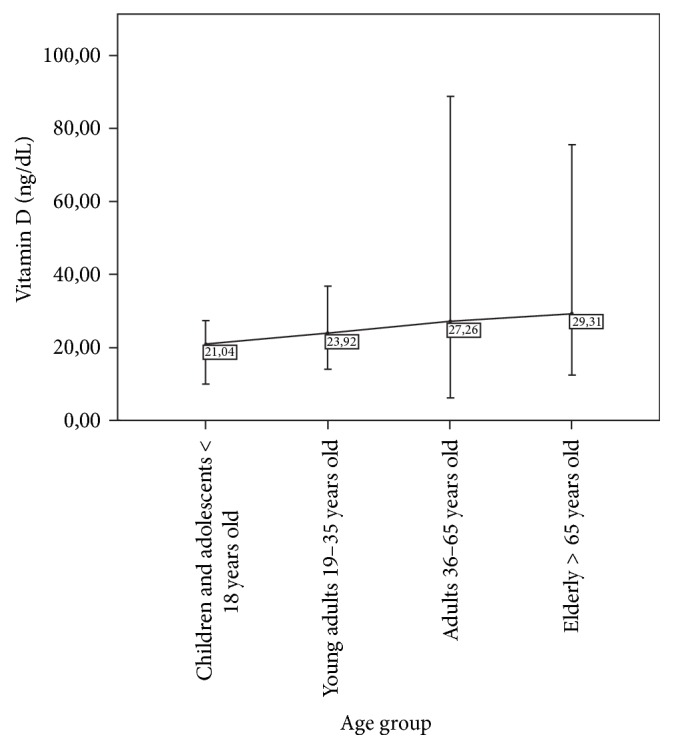
Mean 25 (OH)-D levels in the age groups.

Patients were divided into age groups according to the WHO scale [4] (Table 2) and 25 (OH)-D levels were determined: children-adolescent 21.04 ± 8.01, young adults 23.92 ± 5.48, adults 27.26 ± 10.70, and elderly 29.31 ± 10.65 (Figure 3).

## 5. Discussion

This is a descriptive study of an Ecuadorian population in which levels of 25 (OH)-D, as an indicator of vitamin D status, were determined. 70% of the population showed levels below 30 ng/dl, considered insufficient.

The origin of the population is an important factor when evaluating vitamin D status, because Ecuador is located on the equator at a latitude of −0.95, where the radiation and intensity of UV rays are greater. You would expect the population to have sufficient levels of vitamin D; however, Ecuadorians take measures to avoid sun exposure.

Common sunscreens absorb solar radiation. It has been shown that a sunscreen with a protection factor (SPF) of 30 can absorb up to 95–98% of UV radiation, which reduces the production of vitamin D3 in 95–98% [1]. A study by Matsuoka et al. showed that a sunscreen with an SPF of 8 significantly lowered serum vitamin D3 levels [5, 6]. Also, Matsouka et al. evaluated vitamin D levels in a population of farmers who, because of a significant history of melanoma, took sun protection measures before performing outdoor activities. It was shown that farmers had deficient levels of vitamin D compared to a control group [5].

A study conducted in Atahualpa, a village located on the Ecuadorian coast, determined 25 (OH)-D levels in 220 subjects, of which 25% had levels below 20 ng/ml and was directly related to ischemic events and diffuse subcortical brain damage [7], another reason why the determination and management of vitamin D is essential.

Climatic seasons have a significant influence on the production of cutaneous vitamin D [1]. It has been shown that, in countries with four climatic seasons, vitamin D levels vary. In England, for example, the peak of vitamin D levels occurs in the month of September with an average of 30 ng/dl and the lowest levels occur in February with an average of 14 ng/dL [8]. In cohorts of countries with similar climates like Denmark, vitamin D levels are deficient during the winter months [9]; this data differs from our study, where the mean vitamin D level during the winter was 29.29 ± 3.89 and in the summer, an average of 27.13 ± 3.98. Vitamin D changes in our population are due to the fact that Ecuador consists of two climatic seasons: winter and summer, where UV rays are constant throughout the year (10-11 UV). According to the climatic classification of Köppen [10], Ecuador has a tropical climate characterized by temperatures above 27C with the difference between winter and summer being the amount of fluvial precipitation. Between the months of January and April there are strong and frequent precipitations, whereas in the months between May and December the precipitation is minimal [11].

A study by dermatologists in Australia showed that approximately 87% of Australians after summer had vitamin D levels below 20 ng/dL [12].

Reports from Mexico, Europe, Asia, India, and Africa show that more than 50% of the world's population is at risk of hypovitaminosis D [1, 13, 14], making it a pandemic condition [1, 15]. Data published by the US Centers for Disease Control and Prevention (CDC) show that approximately 32% of children and adults have a significant vitamin D deficiency <20 ng/ml, with hypovitaminosis being more prevalent than obesity in the United States, which is why basic foods such as dairy products were supplemented with vitamin D [12].

As for the age group, vitamin D levels have been shown to be inversely related to age [16, 17], and its production after the sixth decade of life declines [18]. Because of this, supplementation measures are taken in the elderly. In our study, the mean vitamin D level in the elderly group was higher than the other groups. We infer that this is because this group was probably undergoing vitamin D supplementation.

Studies have demonstrated the utility of vitamin D in cardiovascular diseases [19–22], diabetes [23–25], rheumatic and immunological diseases [26–32], hypertension [22, 33, 34], metabolic disorders [35, 36], cancer [37–39], infectious diseases triggered by autoimmune disorders [31], and some neuropsychiatric conditions [40], highlighting the importance of measuring this parameter for a more comprehensive assessment of the patient.

The advantages of our study were that the subjects admitted to the study were not institutionalized, the technique used for the determination of vitamin D was universal, and the origin of the population was exclusively the city of Guayaquil. Within the limitations we consider the lack of sun exposure data and specific measures of sun protection. However, the stated objectives were reached, thus determining that 70% of the population studied showed deficient levels of 25 (OH)-D. More studies of vitamin D status determination are needed in other cities of Ecuador with different geographic characteristics such as the Andes and Amazon region.

## 6. Conclusions

It is evident that despite the location of Ecuador and the intensity of UV rays it receives throughout the year, Ecuadorian subjects have insufficient levels of vitamin D. In the summer months vitamin D levels are higher compared to winter months. Also, vitamin D levels are higher in the elderly compared to other age groups.

## Figures and Tables

**Table 1:** Demographic data and 25 (OH)-D levels.

	*n* = 269	%	*p* ≤ 0.001
Demographic data			
Women	228	85	—
Men	41	15	—
Mean age	54.73 ± 16.58		*p* ≤ 0.001
25 (OH)-D			
Normal	81	30	*p* ≤ 0.001
Deficiency	141	53	*p* ≤ 0.001
Insufficiency	44	16	*p* ≤ 0.001
Severe insufficiency	3	1	*p* ≤ 0.001

Mean	27.29 ± 10.12 [6.41–88.74]		
Women	27.01 ± 9.72 [6.41–75.54]		
Men	28.89 ± 12.18 [11.78–88.75]		

**Table 2:** Age groups.

Age group	*n* = 269	%	*p* ≤ 0.001
Children, adolescents < 18 years old	4	1.5	0.001
Young adults 18–35 years old	44	16.4	0.001
Adults 36–64 years old	133	49.4	*p* ≤ 0.001
Elderly > 65 years old	88	32.7	0.001
